# Supplementary material for: Effects of soil particles and convective transport on dispersion and aggregation of nanoplastics via small-angle neutron scattering (SANS) and ultra SANS (USANS)
Source: PLoS One. 2020 Jul 21;15(7):e0235893. doi: 10.1371/journal.pone.0235893 (PMC7373282; doi:10.1371/journal.pone.0235893)
Supplement: S1 Fig — (PDF) [file pone.0235893.s001.pdf]

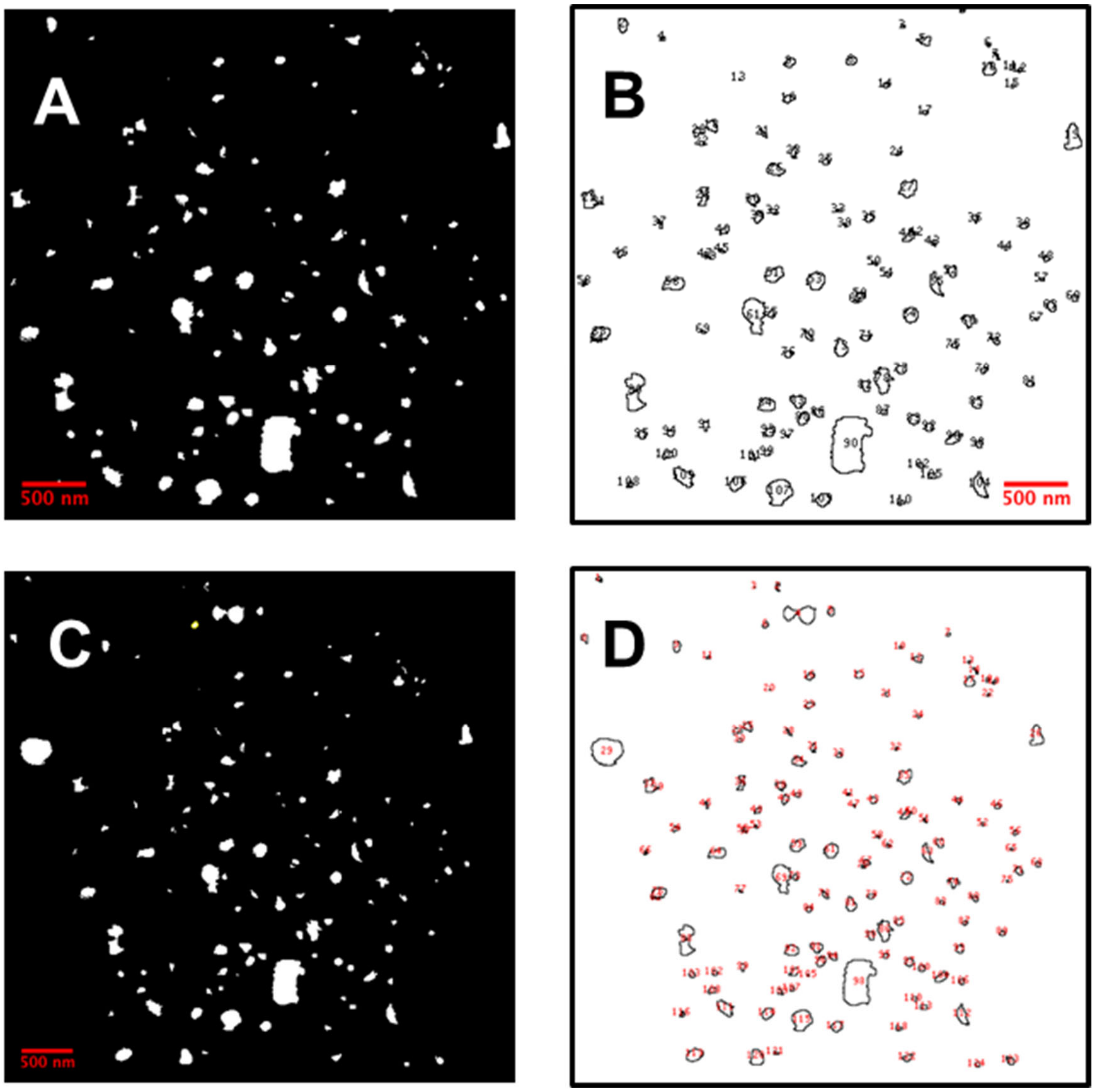

Fig S1. AFM images used for ImageJ measurement of surface roughness for nanoplastics. (A) 400 x 400 dpi and **(C)** 500 x 500 dpi original images. **(B)** and **(D)** show numbering of NPs after the processing of the images for Figs A and C, respectively. AFM images were prepared using a 4 µL aliquot of 5 mg/mL slurry of NPs in water that was deposited on a mica surface and air-dried for 1 h at room temperature (22±1ºC) before scanning. For scanning the samples, a rectangular aluminum cantilever probe composed of an aluminum reflective coating on the backside was used. The AFM images were recorded on a scan area of 5.0 µm × 5.0 µm at a scanning speed of 1 Hz. For ImageJ analysis, the images were adjusted to 100 dpi = 1000 nm.
