# Supplementary figures and images for: Effects of soil particles and convective transport on dispersion and aggregation of nanoplastics via small-angle neutron scattering (SANS) and ultra SANS (USANS)
Source: PLoS One. 2020 Jul 21;15(7):e0235893. doi: 10.1371/journal.pone.0235893 (PMC7373282; doi:10.1371/journal.pone.0235893)

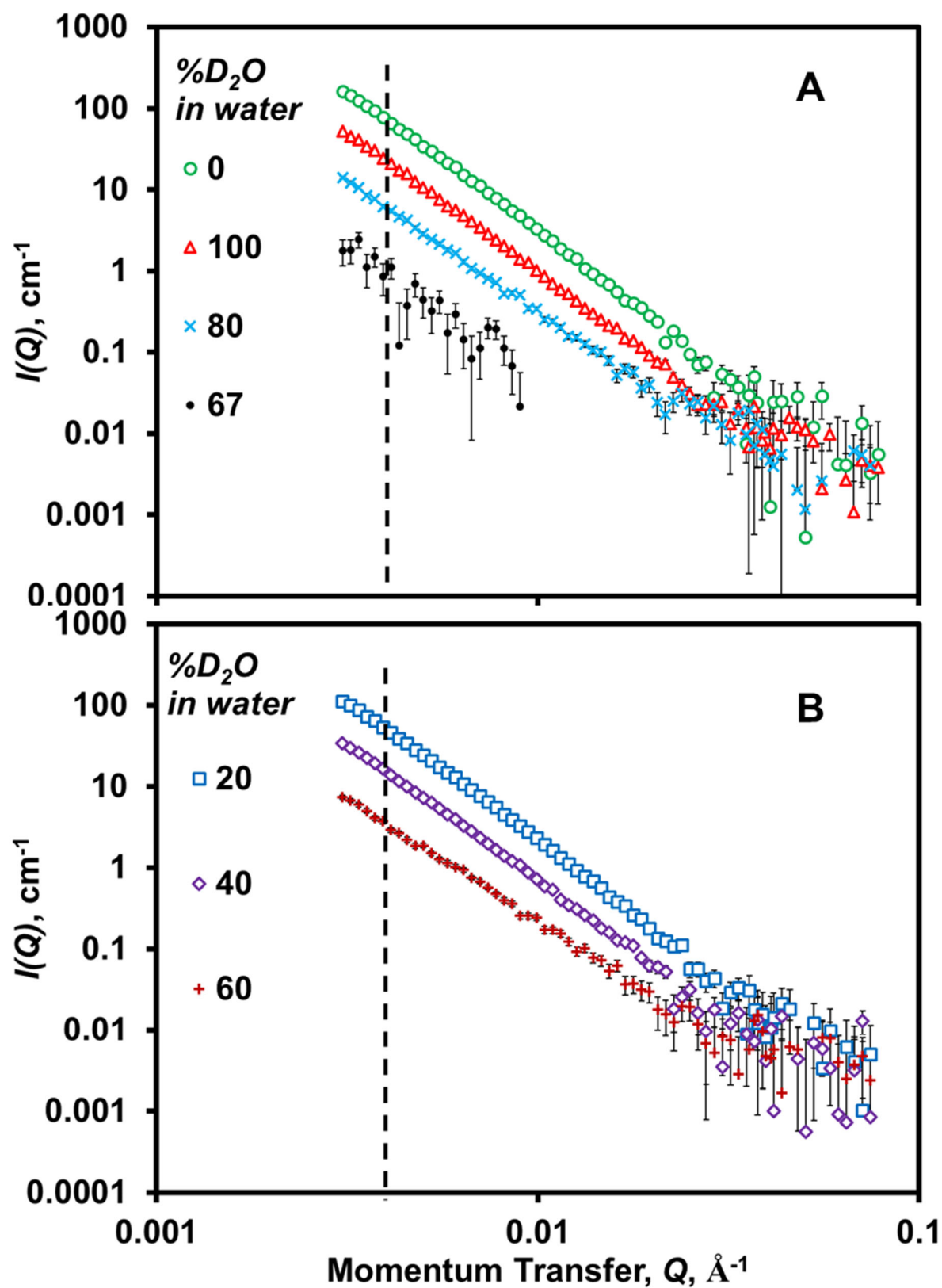

Fig S2. Data of Fig 1A of the main paper replotted to include error bars.

Supplement: S2 Fig — (PDF) [file pone.0235893.s002.pdf]
