# Supplementary material for: Effects of soil particles and convective transport on dispersion and aggregation of nanoplastics via small-angle neutron scattering (SANS) and ultra SANS (USANS)
Source: PLoS One. 2020 Jul 21;15(7):e0235893. doi: 10.1371/journal.pone.0235893 (PMC7373282; doi:10.1371/journal.pone.0235893)
Supplement: Attachment — Submitted filename: Response to reviewers.docx [file pone.0235893.s003.docx]

**19 March 2020**

**Responses to Editor and Reviewers**

*On behalf of my coauthors, I thank the reviewers for their kind assistance in providing us comments and suggestions to improve the manuscript. Our responses are given below.*

*Sincerely,*

*Douglas G. Hayes, corresponding author.*

I. **Responses to Editor (Pratheep K. Annamalai, Academic Editor, *PLOS ONE*)**
1. We have prepared our revised manuscript according to the PLOS ONE's style requirements, including those for file naming.
2. We have revised our Competing Interest Statement to address the involvement of Mobius, Inc. This is provided in our cover letter.
3. We have not made any changes to the Data Availability Statement. We will upload our data as csv files onto Dryad and provide doi numbers for them to PLOS ONE.
4. “Table 3” was a typographical error. “Table 1” should have been used. We have made this correction. Our apologies.
5. We approve of our reviews and responses to the reviewers to be published.
II. **Responses to Reviewer 1**

**Reviewer:** *I thank the authors for this highly interesting and provocative work. I hope that my review, although quite negative, does not discourage these investigations. The work describes small neutron angle scattering over an extended q-range from samples that are to act as proxies for the convective dispersive transport of nano-plastics in soil: vermiculite and a commonly biodegradable plastic mulch. While the sample preparation, and the neutron scattering experiments, their execution and analysis are highly competent, I believe there is a fundamental misconception in applying the findings of this study to the significant environmental transport of pollutants. This is to say the motivation for this study, as put forward in the document’s text is highly questionable. While it has been well described that many environmental pollutants bind to plastic residues and wastes, this property is not unique to these materials, similar binding properties are well known for that ubiquitous colloidal component of soils, humic materials. There is, as far as I understand, not yet, any way to distinguish the transport properties of “manmade” or “natural” soil carbon. This is to ask the question, “what is it that makes binding to nanoplastics different in ecological importance?” I think the way forward in publishing this elegant experimental study is to place a more general emphasis on this preliminary study in an interesting perspective on the transport of soil carbon using contrast variation to selectively visualise this soil component.*

**Response:** We thank the reviewer for their comments. First, we agree with the reviewers that environmental pollutants can bind to soils as well as nanoplastics. Moreover, soils can be apolar when hydrophobic molecules adsorb to soil particulates. However, generally, nanoplastics are more nonpolar than soils. For instance, their water-surface contact angles are typically near 90^o^ (e.g., 82^o^ for the PBAT film we used in our study, as is now reported therein). Second, the particle density of nanoplastics is significantly lower, on average, than the particle density for many soils. As a result, settling velocities of nanoplastics will be lower on average than soil particles.

To address the reviewer’s comments, we have modified the text accordingly (L 89 ff): “.. and differently than soil micro- and nanoparticles (which particularly occur in clays) due to their more hydrophobic nature than most soils. (But, it is noted that adsorption of hydrophobic molecules onto soils can induce hydrophobicity into soils [31].) In addition, the density of NPs for agricultural plastics would significantly lower than soils: 0.5-1 g cm^-3^ for plastics used in mulch films versus particle densities of 2-3 g cm^-3^ for many soils [26, 32].”

**Reviewer:** *Specific comments: I think personally that level of detail in the fitting procedure obscures from the simple messages of this work.*

**Response:** We aimed to keep our description of the SANS and USANS analysis to a minimum, but believed that at least some detail was needed, especially given the diversity of the readership of PLOS ONE. But, to address the reviewer’s comments, we greatly shortened the description of form factor-structure factor modeling by omitting Eq 2-3 and citing a general reference. (top paragr of p. 11)

**Reviewer:** *Page 18 line7:* *Minor typo “NPs were removed from the neutron be through”, neutron beam?*

**Response:** We thank the reviewer for catching this mistake. We have inserted “beam” after “neutron” as recommended (L 262)

**Reviewer:** *Page 18 same paragraph, Ambiguous language:*

*“These results suggest that convection improved dispersion (increased surface area) of the*

*NPs exposed to the neutron beam (e.g., via decreasing the agglomeration of NPs) or increased solvent”*

*Is there some interaction between with the neutron beam which increases dispersion of particles or is something else meant?*

**Response:** We have modified the sentence accordingly: **“***Ex situ* stirring increased the intensity of the SANS signal of PBAT NPs, a result suggesting that convection improved the dispersion of the NPs by disrupting the formation of large agglomerates. An alternate explanation would be that convection increased the extent of solvent penetration into NPs and their agglomerates. The addition of vermiculite reduced the extent of the increase of *I(Q)*.” (L 264-268)

III. **Responses to Reviewer 2**

**Reviewer:** *Dear Authors,*

*Thank you for giving me the opportunity to read your article on nanoparticle dispersion. I found the content has an interesting potential. On the other hand, much more clarification and discussion are required to better understand the content and properly evaluate the true value of the work prior to publication. Especially, Materials and Methods should be further clarified. I suggest this article be completely revised before re-submission for the other review processes. As a conclusion, I recommend its major revision at this state.*

*I hope my comments are helpful.*

*Good luck,*

*A reviewer*

*Major concerns:*

*Materials and Methods: -“apparent density of …g m-2”-> g m-3 ?*

**Response:** Line 123- For films, It is common to express density as “apparent density” in **g m^-2^** . The density can readily be calculated by dividing the apparent density by the film thickness. To address, we have added the following: “(i.e., a specific gravity of 0.787)” (L126)

**Reviewer:** *-“The 45 μm particle fraction was collected, and an average particle size of 38±12 μm…”->Please provide the justification why this size is appropriate for your study.*

**Response:** To address, we added the following sentences: “Soil particles of this size were selected because of their effective dispersion in water, and their high monodispersity was anticipated to simplify interpretation of the SANS data. Vermiculite particles within the given size range mimic silt [33].” (L 138-141)

**Reviewer:** -*How many particles were counted by ImageJ’s “analyze particles” algorithm?*

**Response:** To address, we added the following sentence: “For each particle size fraction, 250 particles were counted and averaged.” (L 146-147)

**Reviewer:**-*“…1.0wt.% PBAT NPs and/or 0.5% vermiculite…”->How did you select these concentrations? Have you also varies them to investigate their effects? According to your introduction, you wish to mimic low concentration of NPs in soil. It seems the particle concentrations are too low, especially for vermiculite to simulate soil. Your samples are like small number of particles swimming in a plenty of water. In agricultural soil, particle movements/displacement should be limited, with much higher volume of soil particles than the one of water.*

**Response:** The authors agree with the reviewer, that the concentrations were lower than would occur in most soils. However, the conditions evaluated would mimic the interface of soils with groundwater. As stated in the Abstract, Introduction, and throughout the manuscript, this study aims to show a proof of concept, if the agglomeration of nanoplastics can be determined in soil via SANS/USANS using neutron contrasting techniques. The employment of low concentrations, 1wt% NPs and 0.5wt% vermiculite, enabled our hypothesis to be tested. We note that we completed SANS measurements within the last 4 weeks that involve a wider range of NP concentration as well as ex situ stirring times. (The SANS experiments were delayed by the unanticipated 1-year shutdown of ORNL’s High-Flux Isotope Reactor.) We will analyze the data and report on our findings later this year, hopefully. We are investigating other soil mimics that will allow us to achieve a stronger contrast match (i.e., *I(Q)* achieved being closer to zero), which will allow us to use higher soil concentrations.

**Reviewer:**- *Do you have any more information about the particle properties (e.g. degree of hydrophobicity, particle roughness)? What is your sample environment (e.g. solution pH and salt type/concentration)? Nanoparticle dispersions can be strongly affected by those factors.*

**Response:** To address, we have included 1) the contact angle of the PBAT-based film (82.5±1.1^o^, indicative of hydrophobicity; L 128; 2) the zeta potential (-22±3.6 mV; L 179); 3) information on the surface roughness (via atomic force microscopy; AFM) and the impact of nanoplastics and vermiculite on pH and conductivity (L 180-176; AFM images provided via the Supporting Information, Fig S1):

“According to Atomic Force Microscopic (AFM) analysis, performed using a model Multimode 8 instrument from Bruker (Santa Barbara, CA, USA), NPs were irregularly shaped and possessed an average roughness of 12.22±1.55 nm (Fig S1 of the Supporting Information).The pH (electrical conductivity) value for the 0.5% vermiculite slurry in water was determined to be 10.14±0.02 (89.57±0.28 µS cm^-1^) and after the addition of 1% NP to the 0.5% vermiculite slurry to be 9.54±0.13 (80.03±0.29 µS cm^-1^).”

**Reviewer:** *-How do you justify your ex situ stirring conditions were appropriate/optimized for your study? Stirring conditions should also affect the particle dispersion/aggregation as well as particle concentrations (see my comments above).*

**Response:** The stir rate of 400 rpm was chosen to allow effective dispersion of nanoplastics and artificial soil in solution. The stir rate was not optimized. As commented upon above, we have very recently investigated a wider range of stirring times via SANS and USANS, and will report on the results in the near future. Stir rate will be investigated in future research.

**Reviewer:***-“…samples were recovered and kept for refrigerated prior to SANS/USANS analysis”->Do you have any proof that this procedure can keep the sample status unchanged? Did you feed your refrigerated sample to a SANS/USANS cell after the sample temperature adjusted to your measurement temperature of 22°C?*

**Response:** Samples after thawing were kept refrigerated at 8°C and adjusted to room temperature 24 hours before measurement. We inserted the following sentence (L 193): “Changes in *d_p_* due to refrigeration were within 5% (DLS analysis).”

**Reviewer:** *-“Typical acquisition times were…8-12 h for…USANS…”->Do you have any proof your sample status does not change during such a long period time? Especially, MP samples might sediment during the measurement.*

**Response:** We cannot verify for certain that size reduction was completely absence during the tumbling of the sample in the neutron beam. But, the tumbling rates used for SANS and USANS were very low (10 rpm and 5 rpm for SANS and USANS, respectively). During preliminary experiments, we found that tumbling rates lower than 5-10 rpm did not produce effective dispersion of particles; therefore, the tumbling rates noted above were used.

**Reviewer:** 2. *Results and discussion:*

*-Figures 1-3: Please add error bars if possible.*

**Response:** We have provided error bars directly to Figs 1B and 3. For Fig 2, error bars are smaller than the symbols; the figure caption was modified to convey this information accordingly. For Fig 1A, the error bars provide too much clutter to the figure. Therefore, we provided the error bars in Fig S2 of the Supporting Information, achieved by dividing Fig A into 2 subfigures. Error bars were also provided for Table 1. An explanation for the error bars of the SANS and USANS data is given in L 211-213.

**Reviewer:** *-Figure 2-A: Please provide the figure legends.*

**Response:** We added the legend to Fig 2A as recommended.

**Reviewer:** *-Figure 2-B: What does “x10, x100, x1000” mean?*

Response: As stated in the caption in both versions of this manuscript, “For Fig B, *I(Q)* was multiplied by a constant (as given in the legend) to improve visualization.”

**Reviewer:** *-“*Ex situ *stirring increased the intensity of the SANS signal of PBAT NPs…These results suggest that convection improved dispersion of the NPs exposed to the neutron beam…”->Does it mean without stirring particles are aggregated and sedimented? If yes, the sample in the beam without experiencing the stirring is not representative to compare with the sample experienced the stirring. If no, why you did not see the change in the scattering curves? Not only the particle numbers, but also particle/aggregate size should also affect the scattering intensity. Referring to your eq.2, the scattering intensity is proportional to (particle volume)2 while (particle number)1.*

**Response:** We are not certain if we fully understand the reviewer’s questions and comments. First, all SANS and USANS samples underwent slow tumbling (5-10 rpm) to ensure that the suspensions were uniform, as described in our responses above. The authors found, that *ex situ* stirring increased the dispersion of nanoplastics, which would increase the surface area of nanoplastics exposed to the neutron beam, and therefore increase *I(Q)*.

**Reviewer:** *-“…the attractive forces between NPs must be weak.”->This statement is vague and do not carry any quantitative information. Consider removing it. If you wish to keep it, please provide your proof. See my comments about the particle properties and sample environments.*

**Response:** We removed the sentence as recommended.

**Reviewer:** *3. Conclusions -“NPs are an emerging threat to soil,…as well as their long-term fate and transport (including to groundwater).”->It sounds like an introduction content. Consider moving and integrating it as a part of introduction.*

**Response:** We believe the sentence should stay in its current position. Moreover, the second paragraph of the Conclusions section provides some perspective of the magnitude of the potential impact of terrestrial nanoplastics and the need for robust approaches to study their fundamental behavior and to detect them in agricultural soils. We believe that SANS+USANS with neutron contrast matching techniques may be particularly valuable to study their fundamental nano-physical chemistry and hope we have demonstrated the method’s potential utility in this paper.

**Reviewer:** *-You may inform possible future work.*

**Response:** We added the following sentence to the end of the Conclusions section: “We are currently evaluating the effect of NP concentration and *ex situ* stirring time on the agglomeration of NPs and soil by SANS and USANS using the approach described herein” (L 352-354).

***Reviewer:*** Minor concerns:

*-Please add line numbers to your manuscript. It makes a reviewer easier to point out his/her concerns.*

**Response:** Line numbers were added

**Reviewer:** *-Title: “…particulates…”->Consider replacing it with “particles” that is used more in the main text. Please be consistent.*

**Response:** “Particulates” was replaced with “Particles” in the title, as recommended

**Reviewer:** *-Introduction: “…plastic plant-soil interactions…”->Do you mean “plastic-soil interactions”?*

**Response:** Line 60- Thank you for catching this. “Plant” was removed

**Reviewer:** *-“…recycling and landfilling…is impracticable due to soil contamination.”->Not sure what you mean. Can you consider rephrasing it or explaining more?*

**Response:** Sentence was modified: “..options. Recycling programs are mostly unavailable [20].” (L 67)

**Reviewer:** *-Figure 3: Define “Schulz polydisperse form factor” in the main text.*

**Response:** The text of the Experimental section now reads:” ‘the polydispersity of the radius (based on a Schulz distribution) *pd* .. [41]” (L229-230). Reference 41 describes the Schultz distribution fully.
