# Supplementary material for: Effects of soil particles and convective transport on dispersion and aggregation of nanoplastics via small-angle neutron scattering (SANS) and ultra SANS (USANS)
Source: PLoS One. 2020 Jul 21;15(7):e0235893. doi: 10.1371/journal.pone.0235893 (PMC7373282; doi:10.1371/journal.pone.0235893)
Supplement: Attachment — Submitted filename: Response_to_Reviewer_2.docx [file pone.0235893.s004.docx]

**20 June 2020**

**Responses to Reviewer 2**

Reviewer #2: Dear Authors,

Thank you for all the efforts. I found that the quality and clarity of your article have been significantly improved. At this point, I have only one additional comment/suggestion below, based on our first exchange. I hope my comments are helpful.
Best regards,
A reviewer

My comments-“Typical acquisition times were…8-12 h for…USANS…”->Do you have any proof your sample status does not change during such a long period time? Especially, MP samples might sediment during the measurement.
Your Response: We cannot verify for certain that size reduction was completely absence during the tumbling of the sample in the neutron beam. But, the tumbling rates used for SANS and USANS were very low (10 rpm and 5 rpm for SANS and USANS, respectively).

->I was talking about aggregation under the very low agitation during the long USANS measurement. Since you cannot separately evaluate the effect of secondary particle addition on the change in particle/aggregate size and associated scattering intensity from the effect of low agitation, I would suggest that you state this point briefly as an indication of the method limitation and for future improvement, for you and other researchers.

**Response:** To address the reviewer’s concern, we added text to the Experimental section:

- L 201-202: “Moreover, the tumbling speed was set to match the settling velocity of particles to ensure that the particles remain mostly in the path of the beam, rather than settle out.”
- L212-215: “We did not observe the settling out of particles at any instance during the SANS or USANS experiments. Although we cannot fully rule out that particle aggregation was induced by low *in situ* tumbling, the absence of settling gives us confidence that the impact of this event was small.”
